# Educate to transform: An innovative experience for faculty training

**DOI:** 10.1007/s10639-022-11160-y

**Published:** 2022-08-01

**Authors:** Noemy Martín Sanz, María Dolores Vivas Urías, Leire Nuere Salgado, Noelia Valle Benítez, María Consuelo Valbuena Martínez

**Affiliations:** 1 Learning & Teaching Department, Universidad Francisco de Vitoria, Ctra. M-515, Pozuelo-Majadahonda km. 1,800, 28223 Pozuelo de Alarcón, Spain; 2 Educational Innovation, OPENUAX, Alfonso X El Sabio University, Avenida Universidad, 1, 28691 Villanueva de la Cañada, Madrid Spain; 3 Online Learning Department, Universidad Francisco de Vitoria, Ctra. M-515, Pozuelo-Majadahonda km. 1,800, 28223 Pozuelo de Alarcón, Spain; 4 Institute of Educational Innovation, Universidad Francisco de Vitoria, Ctra. M-515, Pozuelo-Majadahonda km. 1,800, 28223 Pozuelo de Alarcón, Spain; 5 Active Listening Center, Universidad Francisco de Vitoria, Ctra. M-515, Pozuelo-Majadahonda km. 1,800, 28223 Pozuelo de Alarcón, Spain

**Keywords:** Professor training, Digital resources, Teaching&learning, Faculty, Transformation, Teaching innovation, Educational technology

## Abstract

Learning-focussed educational models require the development of pedagogical, methodological, assessment and technological competences among the faculty community. The COVID-19 pandemic has accentuated the need for this training. This study evaluates the impact of the training project “Educate to Transform” on teacher attitudes, knowledge and on the implementation of innovative teaching methodologies. There were 695 faculty participants in the program conducted at the Universidad Francisco de Vitoria (UFV). Participants included full-time professors (FULL-PROF) and part-time professors (PART-PROF). The measurement instrument was validated using the entire sample and a subsample of 357 participants was used to analyse the impact of the program (pre and post measurement). Professor attitudes and knowledge of or familiarity with innovative methodologies and their application in the classroom were evaluated. The findings show that the program improved the attitudes of teachers towards innovation, raised the level of awareness and number of methodologies implemented in the classroom. The methodology towards more experiential and collaborative learning is effective in transforming teaching practice. Furthermore, the implementation of the program through the CANVAS platform, making teachers live the experience as learners, seems to have contributed to improve the teachers' attitude towards the LMS. The only difference found among the participants was a worse attitude towards innovation on the part of medical teachers, with a clearly differentiated profile of teachers and students, as well as a greater implementation of active methodologies by teachers with a lower teaching load. Overall, it may be concluded that the program achieved its proposed objectives.

## Introduction

Professors, as the activators of student learning and a fundamental element in achieving significant change in the quality of higher education (González-Sanmamed et al., 2020; Madinabeitia & Lobato, 2014), are at the centre of transformation (Trujillo et al., 2020). The professional training and development of educators is a key aspect in achieving this change. The Society for Teaching and Learning in Higher Education (2014) identifies nine ethical principles in university teaching; the first of these is competence in the subject matter, followed by pedagogical competence and student autonomy. However, current practice in university education does not ensure that professors are provided with the pedagogical skills and training necessary to meet the challenges of higher learning (Montes & Suárez, 2016). The educational methods applied by professors are most often derived from their own experiences and a series of stable beliefs, practices and attitudes towards their students (Zabalza et al., 2014). The principal obstacles to pedagogical change are the belief in the transmission of content in which the student acts as a passive receptor and reproducer of information (Krzemien & Lombardo, 2006); the belief that the student has no prior ideas which condition and contextualise their learning; the notion of knowledge as a complete, imputable and encapsulated product; and the acceptance of a broad and superficial curricula with mainly propaedeutic objectives (Garmendia et al., 2014). It is therefore necessary to institute systematic training programs for university professors to develop the pedagogical, methodological, assessment and technological competences necessary to implement learning-centred educational models (Fernández-March, 2006).

The globalised, technological society of the twenty-first century requires digitally fluent professors (Hodges et al., 2020) with specific competences to put technology at the service of a pedagogical model and introduce it into the classroom to enhance the quality of teaching. From this perspective, the teacher and their classroom practice are a central and determining factor in the education of students in a cultural context dominated by digital technologies (Colás Bravo et. al., 2019). Within this scenario, Gallego et al. (2019) and Engen (2019) take a holistic approach to the issue of professors digital competence which includes technological, pedagogical, ethical and attitudinal aspects that permit the appropriate and effective integration of digital technologies into the curriculum and the classroom and thus ensure the digital competence of students (Redecker & Punie, 2017). Miralles et al. (2019) propose an intervention in teacher training based on the theory of Technological Pedagogical Content Knowledge (T-PACK) (Koehler & Mishra, 2008), for an education based on the acquisition of competences using active learning methods (Fernández-March, 2006; Silva & Maturana, 2017), established a direct relation between the implementation of new methodologies and the use of innovative strategies and approaches; a change in the epistemological model of knowledge and the development of digital competences.

Institutions of higher learning (HE) tend to address the professional development of their professors from a conventional perspective, with short training courses that are insufficient and offer limited opportunities for the exchange of experiences (Vu, 2019; Montes & Suárez, 2016, Garmendia et al., 2014; Caballero, 2013). Many researchers have argued for teacher training to be based on teacher experiences and the realities of their professional future, experiential learning and context (Ruiz et al., 2019; Vu, 2019; McCall & McCauley, 2014), engaging in a critical refection on their own teaching practices (Edelstein, 2011; Santos & Sarceda, 2017). The challenge, therefore, is to develop effective strategies of formal and informal professional development (Romeu et al., 2020) empowering professors, incorporating collaborative practices, group tutorials, accompaniment and the transfer of best practices (Vu, 2019; Madinabeitia & Lobato, 2015; Garmendia et al., 2014). The current digital environment offers a multitude of opportunities for learning (formal, informal and self-directed) and a number of studies have been conducted into the notion of Learning Ecologies (LE) as an effective framework. Romeu et al. (2020) note how professors deploy organised systems of activities, relations and resources which can be characterised as components of their learning ecologies which can be continuously updated. Professors’ awareness of the elements of their learning ecologies can enhance their management of the learning process according to their needs, interests and potentialities (González-Sanmamed et al., 2020).

The global health crisis caused by COVID-19 has forced universities to address the challenge of digital transformation head-on. The COVID-19 pandemic affected the educational sector throughout the world to go online (Ferdig & Pytash, 2021; Misirli & Ergulec, 2021; Ryberg, 2021; UNESCO, 2020) and to adjust to the infrastructural and pedagogical requirements of ERT (Emergency Remote Teaching) practice (Barbour et al., 2020, p.6 Hodges et al., 2020). Most of the articles that report on the outcomes in the educational context during the pandemic show the quick leap from face-to-face to online, as well as the difficulties encountered. School teachers were expected to redesign their programmes to support their students in a 100% online environment. In many cases teachers did not possess knowledge about online pedagogies or how to support online learning, as this is not included in many teacher training programmes (McAllister & Graham, 2016). This required a full shift in their pedagogical approach to teaching and learning, and the use of a range of new technologies (e.g., Gurley, 2018). Despite the enormous efforts made by educational institutions to adapt their educational content, activities and assessment systems designed for classroom learning to remote learning, in many cases universities lacked the adequate infrastructure, and also professors lacked the digital competences and aptitudes necessary to adapt to this change (Baladrón et al., 2020; Pardo & Cobo, 2020; Ramírez et al., 2021; Scherer et al., 2021). Online teaching requires technological skills, but also different pedagogical approaches than face-to-face teaching (Gurley, 2018). Teachers need to know which digital online technologies to use and which types of tasks should be included in online learning (asynchronous discussion tasks, online research tasks, video lectures or live video discussions). Regarding online teaching, these learnings may include how to provide clear instructions, how to communicate and assess learning in an effective way.

With the pandemic, the decade-long trend towards online learning has suddenly become a top priority (Burgos et al., 2021; Muzaffar et al., 2021). Studies agree that, after the pandemic, higher education will never be the same again. Change is likely to be on-going, given the need to be prepared for similar events in the future, but there is no consensus on which aspects will be affected and how they will benefit from it. Also, there isn’t a consensus on what model should be followed (Ewing & Cooper, 2021; Howard et al., 2021; Kalantzis & Cope, 2020). For Pardo and Cobo (2020), the current situation should represent a definitive point of departure for the consolidation of processes of innovation in remote learning-teaching, normalise digital culture in higher education and eliminate the barriers between the presential and virtual. This integration of technology into teaching practice facilitates collaborative work (Ali, 2020; World Bank, 2020), providing a new relational framework between teacher and students and among students themselves. Other research findings revealed that, for teachers, the key to leading well-functioning digital classrooms was to develop personal relationships with their students (Olofsson et al., 2021). It is important to foster these educational relations or interactions because they serve to construct the experiences which determine the manner in which students learn. Significant interactions between students and teachers facilitate further reinforcement of knowledge through more in-depth interpretations of concepts, terms and ideas (Thurmond & Wambach, 2004). Furthermore, this affects the learning and modelling of cognitive, affective and psychomotor skills among students (Tirri & Kuusisto, 2013). Managing the emotional engagement of students is one of the most powerful tools in successful outcomes, connecting with student through their personal relations has a significant influence on cognitive processes such as attention, motivation and memory (Burgos et al., 2021; Darby, 2018).

Several studies have explored teacher characteristics that can be associated with the implementation of online teaching and learning (for an overview see Phan & Dang, 2017). Strong leadership and clear support to integrate new technologies and practices in teaching and learning can motivate teachers to change, while a lack of commitment to change at an organisational level can demotivate teachers and limit change (Howard, 2019). To support online learning, several aspects of institutional support, including the schools’ vision and professional development for online teaching need to be addressed. Studies that address teacher profiles and their willingness or unwillingness to accept change also show teachers' perceptions of the readiness of the educational institution, finding a relationship between the acceptance of change and the readiness of the institution. Howard et al. (2021) explore teachers’ perceived readiness to shift their teaching from face-to-face to fully online in response to the COVID-19 pandemic. Readiness is examined in relation to perceptions of how well they felt they were prepared for this change and how well they felt their institutions were prepared. Research has shown that both individual and institutional factors influence teachers’ capacity to take up new digital practices (e.g., Ertmer & Ottenbreit-Leftwich, 2010; Howard, 2019). Therefore, they argue that it is necessary to consider teachers’ perceptions of both their own readiness and that of their institution, to gain a full view of their position. This combined approach can provide a clearer picture of what kind of support is needed, either in terms of training or school agenda setting, to support the transition to online teaching. Teachers’ perceptions of institutional support and related goals and vision in view of online education will have an impact on their overall conception of readiness and ability to teach online (Howard et al., 2021; Philipsen et al., 2019).

For Khlaif et al. (2021) teachers’ capacity-building is not at all an easy task since time and effort are required to empower them with the needed skills. Teachers’ capacity-building in technology and pedagogy should be empowered through training programmes at different levels. It will be an open issue and a major challenge if working with less skillful teachers, which will lead to the continuity of the education system being threatened during different crises. Flexibility is a key issue with teachers’ practices, so when choosing their technological tools a guide should help them to use the suitable one that can be used easily by them and their students (Huang et al., 2020). It is recommended to consider this situation as an opportunity for changing teachers’ classroom practices and developing their knowledge and skills to be ready to engage effectively in online learning. More efforts should be given globally to help developing countries to overcome the issues of digital literacy, digital justice and equity in teacher professional development.

The experience of the COVID-19 pandemic, therefore, can serve as a good reason for the renewal and development of teaching and learning in the university context. Relevant research in the field of university pedagogy has so far highlighted this need (Plota & Karalis, 2019; Raikou, 2019), nevertheless, it is confirmed and given prominence by the current crisis. After all, universities are meant to advocate research, progress and development, therefore the emergence of University Pedagogy as a new scientific field in education is timely and can make a decisive contribution.

Has the COVID-19 pandemic irreversibly changed HE, or instead after the emergency will everything go back to the previous situation? In other words, is the “traditional” HE system going to go back to normal (only in-class activities) or to a “new normal”, characterized by online teaching or in-class teaching that are also video-recorded and/or streamed? This question is part of a wider debate on whether the world and society will be able to take advantage of the changes that have occurred during the COVID-19 crisis with regard to the reduction in greenhouse gasses and waste emissions, and, more generally, towards a more sustainable future (Sarkis et al., 2020).

Furthermore, the “traditional” Universities should not become an alias of the current online (or telematic) Universities, but rather exploit the digital technologies while keeping a strong link between research and teaching activities and the unique on-campus student experience. This is particularly true in technical-scientific fields, in which the face-to-face practical and research activities (in laboratories, field sites, as well as companies) are particularly important. Therefore, traditional Universities should take advantage of the experience gained during the COVID-19 emergency to reshape the content as well as the didactical methodologies of their study programs in order to meet changing students’ needs.

In order to be ready for this “new” normality and to succeed in digital transformation (Uni 4.0), Universities – but also more generally countries and policy makers – should consider the key aspects discussed in Section 2 (perhaps in a different order). First, what are the knowledge and competences that should be taught to students and how can this be done, also exploiting the digital technologies (pedagogical issues)? Second, how should the teaching activities be re-organized to exploit these technologies (e.g., videorecording of lessons, asynchronous lessons for the theoretical part of the courses and then only exercises, discussion of case studies, and laboratory activities done in-class)? Third, what is the best software to support the implementation of the pedagogical and organizational aspects defined in the two previous points? Fourth, is the country system ready from a technical/infrastructural point of view (internet connection, number of laptops per person) for massive online education or blended learning? In many countries there are projects for the development of the broadband network infrastructure (based both on optical fiber and on 5G).

The COVID-19 pandemic has returned us to some of the core questions underpinning systems of HE, such as what are universities for (Collini, 2012)? What do we lose and gain in the shift to online education? Who does the space of the university belong to, how does it foster a sense of community and what is the role of the body in processes of learning, knowing and teaching? Whilst these questions are not new and online education already formed part of the landscape of HE prior to the pandemic, the Covid-19 crisis has brought about fast-paced shifts and has exacerbated social injustices in HE. By re-evaluating our answers to these questions, it becomes possible to take the crisis as an opportunity for reflection (Arendt, 1961, 174), which may lead to reconceptualizing the post-coronial university as a more inclusive, just and equitable institution.

Especially in times of crisis, when the existential questions around the purposes and practices of education become re-emphasized, the act of re-imagining educational utopias is a necessary exercise.

Some of the challenges identified and highlighted by many researchers are summarized as follows: Broadly identified challenges with e-learning are accessibility, affordability, flexibility, learning pedagogy, life-long learning and educational policy (Murgatrotd, 2020).

Pedagogy available and used for face-to-face learning is not feasible for online learning. Though a range of pedagogy has been devised for online and distance learning, teachers who are technologically backward require proper professional development and training in order to orient themselves towards their students. Authentic assessments and timely feedback are essential components of learning. A very crucial part of online distance learning is the availability of helpful formative assessments and timely feedback to the online learners (Doucet et al., 2020). This is found to be challenging for the educators and the education system.

There does not appear to be any studies that suggest that the educational institution, in addition to reinforcing and improving its infrastructure, should draw up a technological, methodological and pedagogical training plan for its teachers that goes beyond guaranteeing a rapid leap from face-to-face to remote, but that aims to deploy its pedagogical model with guarantees in the new scenario.

### The case of UFV: Educate to transform in community program

The new realities of higher education require the implementation of training programs for professors which take into account the importance of renewing the relation with the student, having a direct and significant effect on the learning process and education in general.

This paper presents a study of the effectiveness of the teacher training program Educate to Transform in Community (ETC) in changing the attitudes of professors at the faculty of Universidad Francisco de Vitoria (UFV) and increasing the knowledge and application of innovative methodologies, tools and resources in teaching practice. The ETC training program was conceived as an opportunity to advance in the educational project of the UFV, with an eye to the uncertainties of the 2020–2021 academic year. ETC is based on the educational philosophy of the UFV within the framework of the 2023 Strategic Plan of the university and was designed and implemented as the necessary roadmap to carry out its mission, the search for truth and goodness for social transformation.

The ETC program is based on encounter as the axis of pedagogical model of the UFV, in which the person is regarded as a being who grows and flourishes in the encounter with reality, with themselves and with others (López-Quintás, 2002; Gonzalez-Iglesias & De la Calle, 2020). From these premises the three dimensions of the pedagogical model of the UFV (UFV, 2021) are developed: Awake—Discover—Decide.

- The first step is to inspire existential considerations in the teacher, thus favouring the creation in the classroom the necessary climate for the formulation and expression of searching questions. In this way, the style of the professor and their teaching strategy affect the mood of the classroom, the participation of the students, their attention levels and understanding and thus taking full advantage of the class (Guevara et al., 2005).
- The next step is to discover the answer as a group using an experiential methodology in a manner that the educator-educatee (Oyarzún, 1976) relation becomes the basis of the educational process. The experience of learning of a student is enriched with the teacher constructs an effective pedagogical link (Gallardo, 2010). These ideas reinforce the relevance of the teacher as the mediator in the development of the academic self-perception of the student (Villarroel, 2001). This involves a reinvention or even reversal of the traditional roles of student and teacher thus strengthening the relation between the two.
- The last step would be to decide. In educational literature there exists the classic distinction between educating and instructing, and several authors agree that the ultimate goal of education is the integral formation of the person. This explicitly leads to an anthropological vision of education. To think again, going beyond the purely technical aspects, of the anthropological framework that gives educating real meaning (Muñoz et al., 2016), to use said framework in order to redesign the course subjects and their teaching in the classroom.

The first steps in the way undertaken with the ETC program affect the development of teaching to then have an impact on the students’ education, and involve the transition from a static and traditional training model to a dynamic model adapted to the current needs (Montes & Suárez, 2016; Fernández-March, 2006) and the essential pillars of the UFV (UFV, 2021).

The main purpose of the study is to test if faculty members can be trained, in a program fully designed online, in elearning and hybrid pedagogy, with the active methodologies and digital resources associated with.

Therefore, it was studied the impact of the ETC educational program on the participants' teaching attitudes, knowledge and practices. It is expected that after the delivery of the program, attitude will improve (hypothesis 1), the number of recognised elements involved in teaching work (methodologies, tools and resources) since its implementation will increase (hypothesis 2), the number of recognised elements involved in teaching work (methodologies, tools and resources) since its adaptation will increase (hypothesis 3), and the number of teaching elements (methodologies, tools and resources) that are applied in the classroom will increase (hypothesis 4). That is, from an improvement in the attitude of lecturers, from an improvement in the recognition of the teaching elements involved, with a double perspective: Implementation and adaptation, and from an increase in the application of them.

Moreover, it is expected to find differences in attitude, knowledge and practices according to the profile of the participants, being these full-time professors (FULL-PROF) and part-time professors (PART-PROF), and of the faculty in which they teach (Polytechnic School, Communication Sciences, Health Sciences, Experimental Sciences, Legal and Business Sciences, Education and Psychology and Medicine).

## Materials and methods

### Participants

The total population of the study consisted of 1.850 people of the Universidad Francisco de Vitoria, consisting of 1.300 full-time professors (FULL-PROF) and 550 part-time professors (PART-PROF).

A total of 695 people participated in the study (Table 1), of whom 482 were FULL-PROF (69.4%) and 213 were PART-PROF (30.6%). The average age of the participants was 45.46 (SD = 9.74), the youngest participant was 23 years of age and the oldest was over 70. By gender, 367 were women (53.1%) and 328 men (46.9%).

**Table 1:** Details of the research

Step	Description	Universe	Total sample	FULL-PROF	PART-PROF
1	Reliability and validity of the survey	Professors	695	482	213
2	Pre-post comparison		357	252	105

The complete sample of 695 people was used in verifying the reliability and validity of the questionnaire.

To analyse the impact of the training program, the sample consisted of 357 participants who answered both the pre and post evaluation. These participants were distributed in the three editions of the program, 61 participants in the first, 110 in the second and 183 in the third. Of the 357 participants, some 252 were FULL-PROF (70.6%) and 105 were PART-PROF (29.4%). The average age of the participants was 46.14 (SD = 9.79), the youngest was 24 years of age and the oldest 68. By gender, 188 were women (52.7%) and 169 men (47.3%).

In terms of the distribution by faculties in which teaching was given, 9.9% were from the Polytechnic School (*n* = 35), 24.6% from Communication Sciences (*n* = 88), 16.3% from Health Sciences (*n* = 58), 11.9% from Experimental Sciences (*n* = 43), 20.6% from Legal and Business Sciences (*n* = 74), 12.3% from Education and Psychology (*n* = 16) and 4.4% from Medicine (*n* = 16).

### Design

This was a quasi-experiment with a pre-post measurement of a single group. The dependent variables were the attitude towards innovation in teaching practice, the degree of knowledge about the implementation and suitability of elements of teaching practice and the degree to which these were applied in the classroom.

### Measurement instruments

#### Attitudes towards teaching practice

To evaluate attitudes an ad hoc questionnaire was created consisting of 6 items on a Likert-type scale from 1 to 6 (Table 2). This consisted of two dimensions; the first dimension was the attitude towards innovation (items 1, 2 and 3), and the second dimension was the attitude towards LMS (items 4, 5 and 6). Item 6 is an inverse item.

**Table 2:** Evaluation of attitude

Item Nº	Statement
Item 1	It is possible for students to learn in spaces other than the classroom
Item 2	The teacher foments not only the acquisition of knowledge but also the development of other transversal skills
Item 3	The teacher promotes the autonomy of the student
Item 4	The virtual classroom is a space that facilitates the learning experience of the student
Item 5	The virtual classroom helps in the relation with my students
Item 6	The virtual classroom hinders collaborative work among students

In line with Casas and Blanco-Blanco (2017) and Abad et al. (2011), the values for reliability (α: 0.750 and ω: 0.850), and the model fit (CFI: 0.987; TLI: 0.967; RMSEA: 0.050 and X2/gl: 2.759), provide empirical evidence of the suitability of the model. Cronbach’s alpha for the dimension innovation was 0.615, and for the LMS dimension was 0.729. Both dimensions explain 62.11% of the variance.

#### Knowledge and application of elements of teaching practice

To evaluate the knowledge and application of elements of teaching practice a scale was created which included three *checklists* that evaluated knowledge on the implementation, adaptation for use in the classroom of 22 elements related to teaching practice and learning activities: 8 teaching–learning methodologies, 5 evaluation instruments and 3 formats of multimedia resources (Table 3). Participants indicated the elements they were familiar with, knew how to adapt to the classroom and which of these they made use of in the classroom.

**Table 3:** Element of teaching practice

Elements of teaching practice
Project Based Learning
Problem Based Learning
Group work
Cooperative learning
Forums
Wikis
Debates
Flipped classroom
Simulation
Gamification
Service learning
Conceptual maps
Oral presentations
Video tutorial
Enriched video
Podcast
Portfolio
Rubrics
Peer to peer evaluation
Closed questions
Open questions
Master class

### Procedure

During June and July of 2020 over 1,000 UFV professors were called to participate in the program “Educate to Transform in Community” with the aim of inspiring, awakening and encouraging a change in the relation with students, beginning with the opportunity to reflect and renovate their own teaching practice. Within the plan for hybrid teaching at the UFV the following objectives were established:

- Looking deeper into the education project of the UFV
- Reflecting on learning objectives and results and on the professor-student relationship.
- Inspiring and discovering the need to innovate in the design and development of the subject taught, by deepening the knowledge of active learning teaching methodologies, a renewed evaluation, and self-diagnostic questionnaires and results measurement.
- Discovering meaningful learning with the LMS Canvas platform.
- Sharing learning with the UFV community, bringing about the redesign of the subjects and their virtual classrooms.

For the planning of the program, four work teams (pedagogical model, innovation, evaluation and LMS) were formed, together with a community of designers and trainers who were responsible for developing and accompanying lecturers in their experience and learning.

The total duration of the program was one month: two intensive weeks of knowledge acquisition in online format, one of tutorials in which to to deepen and reflect on what was learned, and one last week to start to put into practice what had been learned in the assembly of the virtual classrooms of the different subjects. During the months of June and July, to ensure personalised attention and accompaniment of the lecturers, three editions of the program were carried out. In addition, an intensive version (Canvas Express) was designed for new lecturers who joined in September and a reminder course (Canvas Refresh).

**Fig. 1:**
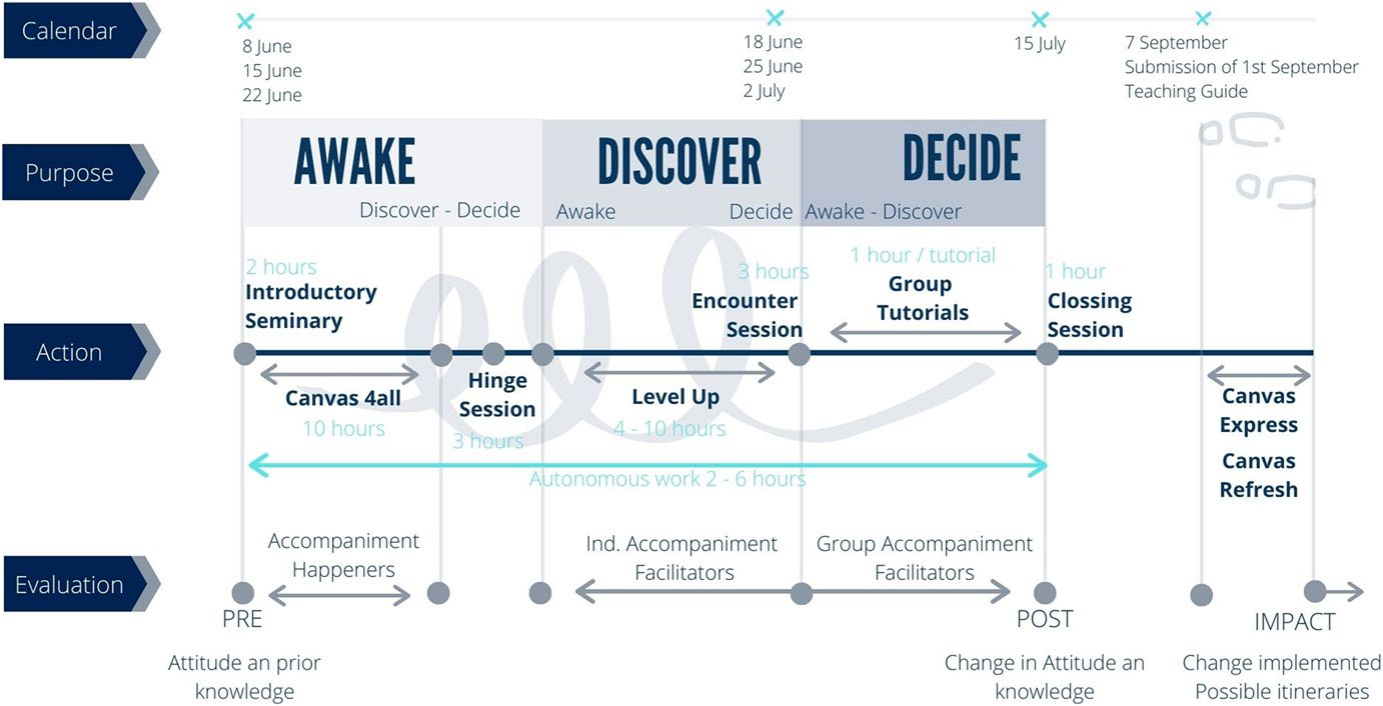
Diagram of the program and calendar of editions. Source: Universidad Francisco de Vitoria (2020)

The program was designed applying the pedagogical model of the university (Awake—Discover—Decide). Figure 1 shows the content and experiences deployed during the various stages of the course:

1. Three synchronised, online points of encounter were established:

- Introductory Seminar: to inspire and contextualise change, four models of the training project were addressed (educational, pedagogical, didactic and curricular), the role of evaluations as part of the learning process, the importance of innovation in teaching practice through different methodologies and the potential of the new platform in virtual learning.
- Encounter Session: to share, in community, the discoveries and skills acquired as well as to foment links between different models and the path travelled thus far.
- Closing Session: a plenary session to summarise, in community, the skills acquired and organise their application to the courses, involving the redesign of courses, teaching guides and virtual classrooms.

2. Canvas4ALL: an asynchronous, gamified online learning experience in which UFV professors were taken on a virtual tour of various countries considered leaders in education. The aim was to share learning experiences and reflection in pairs before beginning the first mission; the professors selected a community which best fit with their interest in the journey (learning facilitator, innovation, renewed evaluation or learning communities). In each of the five stops on the tour professors were presented with a series of missions to overcome with the aim of discovering best practices in teaching innovation and evaluation using the tools provided by the new Canvas platform. Professors were given personalised feedback on the work submitted for each stage.
3. Hinge session: consisting of three sessions designed to guide professors in discovering the educational model of the UFV:

- Canvas as a pedagogical space: best practices which demonstrate the opportunities for the design and generation of experience offered by the Virtual Classroom.
- Pedagogical outlook: this addresses questions such as: what is the goal of my course? What are the desired learning outcomes? What should be the teacher-student relation?
- Formative activities: inviting each professor to rethink science in terms of four broad themes: anthropology, epistemology, ethics and meaning (UFV, 2021, p. 13).
- LevelUP: consisting of a catalogue of 22 asynchronous online seminars to further explore active methodologies, tools and resources to favour effective learning, student autonomy and facilitate the teacher-student relationship. To pass this block professors were expected to develop a proposal for the application of a methodology and/or tool and share it with the rest of the professors and facilitators of the UFV Community.

4. Group Tutorials: synchronous online sessions to analyse in-depth the methodologies and tools that raised the most interest during the course.

To accompany the professors on their journey a community of change agents, expert in new technologies and methodologies was on hand to accompany professor, particularly in Canvas4ALL (Happeners) and LevelUP (Facilitators).

### Data analysis

The reliability was evaluated using Cronbach’s alpha and McDonald’s omega. To verify the fit of the resulting Exploratory Factor Analysis (EFA) a Confirmatory Factor Analysis (CFA) was conducted. The CFA tests the theoretical validity of the model, later contrasted with the data drawn from the sample (Abad et al., 2011). The WLSMV (weighted least square mean and variance adjusted) method was used. Firstly, the chi-squared statistical coefficient was used within its degrees of freedom. The CFI (Comparative Fit Index) and TLI (Tucker- Lewis Index) were used as comparative indices. Finally, two residual indices were obtained, SRMR (Standard Root Mean Square Residual) and RMSEA.

To assess the possible effect of the program edition on the results, a single factor ANOVA was conducted. To verify the hypotheses a Student’s t test was conducted for the related samples.

The IBM SPSS v25 program was used for Cronbach’s alpha, EFA and ANOVA. The AMOS Graphics v24 program was used for CFA, and the RStudio v1.3 program was used for McDonald’s omega.

## Results

### Comparison of editions

Before beginning with the verification of the proposed hypotheses, the results of the three editions of the program were analysed and compared to verify that any particular edition did not unduly effect the results. An evaluation of the sample of participants of the three editions show similar values for attitude, both prior to the program (F_2,353_ = 0.636, *p* > 0.05) and after their participation in the program (F_2,288_ = 0.392, *p* > 0.05); regarding knowledge of the implementation of these elements, both prior to the program (F_2,277_ = 0.502, *p* > 0.05) and after (F_2,277_ = 1.929, *p* > 0.05); knowledge of the suitability of these elements, both prior to the program (F_2,277_ = 1.850, *p* > 0.05) and after (F_2,277_ = 0.068, *p* > 0.05); and their practical application in teaching, both prior to the program (F_2,277_ = 1.446, *p* > 0.05) and after participation (F_2,277_ = 1.166, *p* > 0.05).

### Changes in attitude

For the first hypothesis, the improvement of attitudes towards innovation and LMS were evaluated (Hypothesis 1). The results show that there was an improvement both in the general attitudes (t_286_ = -3.519, *p* < 0.01), and towards innovation (t_291_ = -1.982, *p* < 0.05) and the LMS (t_287_ = -3.440, *p* < 0.01) (Table 4).

**Table 4:** Change in attitudes before and after the program

	PRE		POST	
	M	SD	M	SD
Attitude – Total	30.15	3.64	30.99	3.46
Attitude towards Innovation	16.64	1.62	16.84	1.52
Attitude towards LMS	13.51	2.64	14.15	2.65

*M* Mean, *SD* Standard Deviation

In relation to the differences in attitude change between FULL-PROF and teaching PART-PROF, both groups returned similar results in total attitude (F_1.285_ = 0.233, *p* > 0.05) and their dimensions, both in attitude toward innovation (F_1.290_ = 0.147, *p* > 0.05) and toward LMS (F_1.286_ = 0.139, *p* > 0.05), both before and after the program was performed.

As for the differences between faculties, the Faculty of Experimental Studies (M = 32.11, DT = 2.82, *p* < 0.05) and Communication Sciences (M = 31.19, DT = 3.88, *p* < 0.05) have been found to be the faculties that have the best overall attitude after attending the program. This contrasts with the Medical Faculty, which showed the worst attitude (M = 29.18, DT = 3.76). In the attitude toward innovation, it is the Faculty of Legal and Business Sciences that presents a better attitude (M = 17.00, DT = 1.53), compared with the Faculty of Medicine, which presents the worst attitude (= 15.91, DT = 2.30, *p* < 0.05). No differences were found in attitude toward LMS.

### Changes in knowledge of the implementation, suitability, and application of elements of teaching practice

Secondly, the differences in knowledge and application of the elements in teaching practice was analysed. It was found that the number of elements teachers knew how to implement increased after the program (t_277_ = -8.984, *p* < 0.01), with an increase in awareness of their suitability (t_277_ = -8.229, *p* < 0.01) and the number of elements applied in the classroom (t_277_ = -6.983, *p* < 0.01) (see Table 5).

**Table 5:** Changes in knowledge of implementation, suitability and application of elements before and after the program

	PRE		POST	
	M	SD	M	SD
Knowledge of implementation	15.09	4.32	17.34	4.40
Knowledge of suitability	12.88	5.08	15.47	5.40
Application	9.04	3.39	10.41	3.80

*M* Mean, *SD* Standard Deviation

Specifically, in terms of familiarity with implementation differences were found for all elements with the exception of group work, debates, conceptual maps, oral presentations, video tutorials and podcasts. The elements showing the greatest differences were service learning, wikis and simulation (see Table 6).

**Table 6:** Changes in knowledge of elements of teaching practice

Element of teaching practice	Implementation						Suitability					
	PRE		POST		sig	Res.*	PRE		POST		sig	Res.*
	Not	Yes	Not	Yes			Not	Yes	Not	Yes		
Project Based Learning	51	227	27	251	0.001	4.7	77	201	45	233	0.000	5.3
Problem Based Learning	77	201	35	243	0.000	4.2	105	173	47	231	0.000	2.7
Group work	-	-	-	-	0.238	-	-	-	-	-	0.324	-
Cooperative learning	87	191	46	232	0.000	5.1	112	166	59	219	0.000	5.2
Forums	40	238	19	259	0.001	4.2	77	201	59	219	0.044	5.1
Wikis	184	94	147	131	0.000	9.1	211	67	184	94	0.001	8.4
Debates	-	-	-	-	0.105	-	-	-	-	-	0.222	-
Flipped classroom	128	150	48	230	0.000	5.7	150	128	77	201	0.000	7.2
Simulation	137	141	115	163	0.013	8.1	-	-	-	-	0.054	-
Gamification	107	171	54	224	0.000	6.9	142	136	94	184	0.000	6.8
Service learning	211	67	182	96	0.000	10.6	219	59	187	91	0.000	8.7
Conceptual maps	-	-	-	-	0.229	-	132	146	101	177	0.001	6.5
Oral presentations	-	-	-	-	0.061	-	-	-	-	-	1.00	-
Video tutorial	-	-	-	-	0.749	-	-	-	-	-	0.114	-
Enriched video	207	71	110	168	0.000	3.4	217	61	134	144	0.000	3.0
Podcast	-	-	-	-	0.148	-	160	118	134	144	0.009	5.8
Portfolio	148	130	92	186	0.000	6.1	175	103	128	150	0.000	6.3
Rubrics	60	218	23	255	0.000	4.8	89	189	49	229	0.000	5.5
Peer to peer evaluation	109	169	40	238	0.000	4.0	134	144	64	214	0.000	4.0
Closed questions	-	-	-	-	0.087	-	67	211	45	233	0.005	6.2
Open questions	37	241	21	257	0.011	5.5	71	207	49	229	0.008	5.6
Master class	-	-	-	-	0.690	-	-	-	-	-	0.888	-

Regarding knowledge about their suitability, differences were seen in all elements except for group work, debates, simulation, oral presentations, video tutorial and master class (see Table 6). The elements with the greatest increase in knowledge were wikis and service learning.

No significant differences have been found between FULL-PROF and teaching PART-PROF, or between faculties.

Finally, for application, the results show that certain elements and methodologies were applied with greater frequency after the program: Project Based Learning, Problem Based Learning, cooperative learning, flipped classroom and peer to peer evaluation; forums, video tutorials, enriched videos and rubrics were also used with greater frequency (see Table 7).

**Table 7:** Changes in the application of elements of teaching practice

Element of teaching practice	PRE		POST		Sig.	Res.*
	Not	Yes	Not	Yes		
Project Based Learning	133	145	112	166	0.011	9.2
Problem Based Learning	150	128	115	163	0.000	6.3
Group work	33	245	49	229	0.044	3.5
Cooperative learning	161	117	121	157	0.000	5.6
Forums	150	128	121	157	0.004	5.5
Wikis	-	-	-	-	1.000	-
Debates	-	-	-	-	0.625	-
Flipped classroom	206	72	133	145	0.000	7.2
Simulation	-	-	-	-	0.897	-
Gamification	-	-	-	-	0.272	-
Service learning	-	-	-	-	1.000	-
Conceptual maps	-	-	-	-	0.120	-
Oral presentations	-	-	-	-	0.512	-
Video tutorial	155	123	110	168	0.000	5.8
Enriched video	259	19	213	65	0.000	4.2
Podcast	-	-	-	-	0.327	-
Portfolio	-	-	-	-	0.222	-
Rubrics	111	167	89	189	0.015	6.9
Peer to peer evaluation	230	48	178	100	0.000	5.9
Closed questions	-	-	-	-	1.000	-
Open questions	-	-	-	-	0.539	-
Master class	-	-	-	-	0.253	-

In the case of group work the difference before and after the program was also significant; however, fewer professors used this methodology after the program than before.

In relation to differences between profiles, the results show that teaching PART-PROF (M = 11.21, DT = 4.05) apply more methodologies after the course of the program than FULL-PROF. (M = 10.07, DT = 3.62) (t_266_ = 2.20, *p* < 0.05). No differences were found between faculties.

## Discussion and conclusions

The results of the study show that the training program met the proposed objectives and hypotheses. After the implementation of the program there was an improvement in attitudes towards innovation (t_291_ = -1.982, *p* < 0.05) and LMS (t_287_ = -3.440, *p* < 0.01) (hypothesis 1), the university professors reported that they were familiar with how to implement (t_277_ = -8.984, *p* < 0.01) (hypothesis 2), the suitability (t_277_ = -8.229, *p* < 0.01) (hypothesis 3) and the application (t_277_ = -6.983, *p* < 0.01) (hypothesis 4) of a greater number of active methodologies, tools, evaluation instruments and resources for teaching practice. Furthermore, the elements of teaching practice where there were changes, such as Project Based Learning, Problem Based Learning, cooperative learning and flipped classroom, are student-centred, making the student the active agent in the learning process. This transformation in the attitude of professors with greater familiarity and application of active methodologies, evaluation instruments and multimedia resources, will most likely have a positive effect on the quality of the teaching–learning process. And also, in its ability to design face-to-face courses with the support of technology, such as instructional, pedagogical and didactic design for online or hybrid courses, with active learning methodologies and adequate digital resources and applied in accordance with e-learning quality standards. Facilitating, in turn, the scalability in the implementation of new technologies, thanks to the best digital skills of teachers.

The faculties with the best attitude were the Faculty of Experimental Studies (M = 32.11, DT = 2.82) and Communication Sciences (M = 31.19, DT = 3.88), while the worst attitude was found in Medical Faculty (M = 29.18, DT = 3.76). A possible explanation for the differences found between faculties may be due to the different dynamism and participation in the course. This result could be due to the small number of the sample and to the fact that most of the teachers were external (practicing physicians, with less time for training as teachers and therefore less receptive to change). On the other hand, the profile of medical students, who are very focused on passing a qualification exam, are less receptive to teaching changes, perhaps generating a subjective reluctance on the part of their teachers.

The difference between the FULL-PROF (M = 10.07, DT = 3.62) and PART-PROF (M = 11.21, DT = 4.05) in the implemented methodologies could be explained because may be due to a greater resistance to the pedagogical change of FULL-PROF, due to its high workload. While the PART-PROF, having less teaching load, perceive themselves as more available and hence have a better attitude. No differences have been found in terms of knowledge because knowing in both cases comes in the same way, innovation days, dissemination, congresses… the difference is found when applying it in the classroom.

In addition to the effect on professors, the design of the training program and its implementation reveal aspects of training that should be taken into account for future actions in teacher training. The results show that the change in methodology towards more experiential and collaborative learning, even in an asynchronous online format, is effective in the transformation of teaching practice. The implementation of the program through the CANVAS platform appears to have helped improve the attitude of professors towards LMS, as their own experience as students using the platform was an opportunity for them to experience first-hand the potential of the platform.

It is also a very valuable contribution that the design of training under an experiential methodology, which has made them live the experience of their students in first person, totally changes the teacher's mindset when designing their courses in the virtual classroom. On the other hand, the methodology and the platform used allow the program to have scalability, being able to reach a large number of teachers without making large investments.

Future lines of research should explore further the causes of the lack of transfer to teaching practice those methodologies in which there was increased awareness both in terms of application and suitability, as was the case with service-learning. In this case, a possible hypothesis may be the difficulty in implementing this methodology is due to the need for structural resources (Romeu et al., 2020). Future research could also, in line with the study by García-Martín and García-Sánchez (2017), explore the relation between the implementation of active methodologies and the use of innovative strategies and approaches with the development of digital competences on the part of professors. This could help determine if differences in the effectiveness of training program depend on the digital competence of professors (Miralles et al., 2019; Scherer et al., 2021).

An intelligent analysis of the qualitative information gathered in the training program, learning diaries and action plans submitted by teacher in LMS using Natural Language Processing and Machine Learning, along with self-classification data of teacher by characteristic profiles regarding their attitudes towards experiences and life, will permit the creation of a map of attitudes and aspirations from a new perspective that can aid the development of personalised training programs. This analysis would also contribute to a more complete and consolidated vision of the outcomes of the training programs.

One of the limitations of the study is the use of a single source of assessment such as lecturer perception (Madinabeitia & Lobato, 2015) that, in order to improve diagnosis and evaluation, should be supplemented with other evidence such as LMS platform indicators. The perception of the student, the lecturer's teaching work levels, seniority in the position, etc. The analysis of this data would allow us to undertake further research such as the analysis of lecturer profiles. Another limitation of the work is the moderate level of lecturer participation in the survey, which could indicate a bias in the response, as those who have answered might be those who have the greatest motivation and interest in the program.

## Data Availability

Materials and data are available if requested by the journal.
